# Domain III of Cry1Ac Is Critical to Binding and Toxicity against Soybean Looper (*Chrysodeixis includens*) but Not to Velvetbean Caterpillar (*Anticarsia gemmatalis*)

**DOI:** 10.3390/toxins10030095

**Published:** 2018-02-27

**Authors:** Rubina Mushtaq, Abdul Rauf Shakoori, Juan Luis Jurat-Fuentes

**Affiliations:** 1 School of Biological Sciences, University of the Punjab, Quaid-i-Azam Campus, Lahore 54590, Pakistan; rubina.sbs@pu.edu.pk; 2 Department of Entomology and Plant Pathology, University of Tennessee, Knoxville, TN 37996, USA

**Keywords:** *Bacillus thuringiensis*, Cry1Ac, *Chrysodeixis includens*, *Anticarsia gemmatalis*, toxin domain

## Abstract

Insecticidal proteins Cry1Ac and Cry2Ac7 from the bacterium *Bacillus thuringiensis* (Bt) belong to the three-domain family of Bt toxins. Commercial transgenic soybean hybrids produce Cry1Ac to control the larvae of the soybean looper (*Chrysodeixis includens*) and the velvet bean caterpillar (*Anticarsia gemmatalis*). The specificity of Cry1Ac is determined by loops extending from domain II and regions of domain III in the three-dimensional structure of the toxin. In this study, we constructed a hybrid toxin (H1.2Ac) containing domains I and II of Cry1Ac and domain III of Cry2Ac7, in an attempt to obtain a protein with enhanced toxicity compared to parental toxins. Bioassays with H1.2Ac revealed toxicity against the larvae of *A. gemmatalis* but not against *C. includens*. Saturation binding assays with radiolabeled toxins and midgut brush border membrane vesicles demonstrated no specific H1.2Ac binding to *C. includens*, while binding in *A. gemmatalis* was specific and saturable. Results from competition binding assays supported the finding that Cry1Ac specificity against *A. gemmatalis* is mainly dictated by domain II. Taken together, these distinct interactions with binding sites may help explain the differential susceptibility to Cry1Ac in *C. includens* and *A. gemmatalis*, and guide the design of improved toxins against soybean pests.

## 1. Introduction

Cry proteins from the bacterium *Bacillus thuringiensis* (Bt) are used in sprays or produced by transgenic crops for environmentally friendly pest control [1]. While diverse, the vast majority of Cry proteins present a shared structural three-domain organization, supporting a similar mode of action. The Cry1Ac toxin is one of the most active Bt toxins against the larvae of Lepidoptera. The steps involved in the mode of action of Cry1Ac and other three-domain Bt toxins have been thoroughly reviewed [2]. After ingestion, the Cry1Ac toxin is processed in the midgut fluids to an active core that recognizes binding sites in proteins in the midgut brush border epithelium. This binding is conducive to further processing of the toxin and formation of an oligomer, which inserts in the membrane of midgut cells to form a pore through the insertion of amphipathic alpha helices in domain I. The toxin pore leads to osmotic cell death, collapsing of the midgut epithelial barrier, and invasion by resident midgut bacteria of the hemocoel to cause septicemia and the death of the insect.

There is substantial evidence from mutagenesis studies supporting the view that the critical toxin-binding step of Cry1 toxins is specified by interactions between domains II and III with midgut receptors [3]. Construction of hybrid Cry toxins through domain substitution has allowed the identification of domains relevant to binding specificity, and in some cases in hybrids with increased toxicity and expanded target range compared to parental toxins [4,5,6]. For instance, hybrids composed of Cry1Aa and Cry1Fb proteins displayed increased activity against *Agrotis ipsilon* [7]. Domain shuffling has identified the relevant role of domain III in toxicity. A hybrid containing domains I and II of Cry1Ab with domain III of Cry1Ca displayed increased toxicity against the larvae of *Spodoptera exigua* when compared to parental toxins [8]. Similarly, domain III of Cry1Ac increased activity in hybrid toxins against *Heliothis virescens* larvae [9], while combining domains I and II of Cry1Ac and domain III of Cry1Ca resulted in hybrids with novel specificities compared to the parental toxins [10]. Domain III shuffling between members of diverse Cry toxin families has also resulted in hybrids with improved activity. For example, the eCry3.1Ab hybrid containing domains I and II of the coleopteran-active Cry3Aa toxin and domain III of the lepidopteran-active Cry1Ab toxin presented increased toxicity against the larvae of *Diabrotica virgifera* [11].

In this work, we aimed to test the importance of domain III in the toxicity of Cry1Ac against *Chrysodeixis includens* (soybean looper) and *Anticarsia gemmatalis* (velvetbean caterpillar), as this domain contains a lectin fold involved in binding to N-acetylgalactosamine (GalNAc) on putative receptors [4]. Larvae of *C. includens* and *A. gemmatalis* are the most economically relevant pests of soybean on the American continent, and transgenic soybean (*Glycine max*) producing the Cry1Ac protein was commercialized to control these larvae [12]. We report testing of a hybrid gene (H1.2Ac) containing domains I and II of Cry1Ac and domain III of Cry2Ac7, which is a novel toxin active against both *C. includens* and *A. gemmatalis* [13]. Bioassay and binding tests with the generated H1.2Ac hybrid support that domain III is critical for Cry1Ac toxicity against *C. includens*, but not to *A. gemmatalis*.

## 2. Results

### 2.1. Hybrid Protein Construction and Production

**Figure 1:**
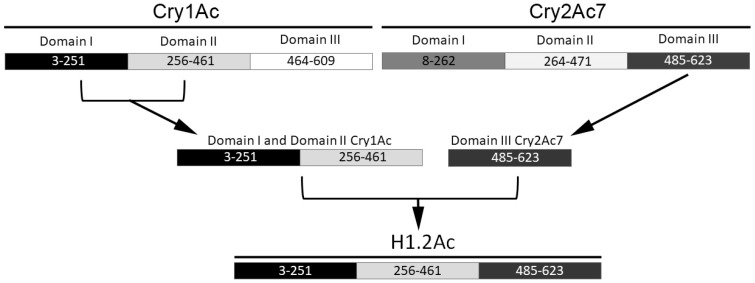
Schematic representation of the construction of hybrid protein H1.2Ac. The bars represent the polypeptides; different color indicate different domain segments of each protein, as indicated. Numbers indicate amino acid residues.

Domains I and II of the trypsinized form of Cry1Ac spanned from amino acid residues 3–251 and 256–461, respectively, and domain III encompassed amino acids 464–609 (Figure 1). This third domain of Cry1Ac was substituted with domain III of Cry2Ac7, which spanned from amino acids 485–623 (Figure 1). Successful cloning of the hybrid protein, which was named as H1.2Ac, was verified by restriction analysis and DNA sequencing. The recombinant H1.2Ac gene (1809 bp) encoded a protein of 602 amino acids with a predicted molecular weight of 67.9 kDa. In silico analysis of the H1.2Ac protein using protein signature databases through InterProScan identified domain I to include amino acid residues 3–251, while amino acid residues 256–461 formed domain II, and amino acid residues 463 to 602 formed domain III (Figure 1).

**Figure 2:**
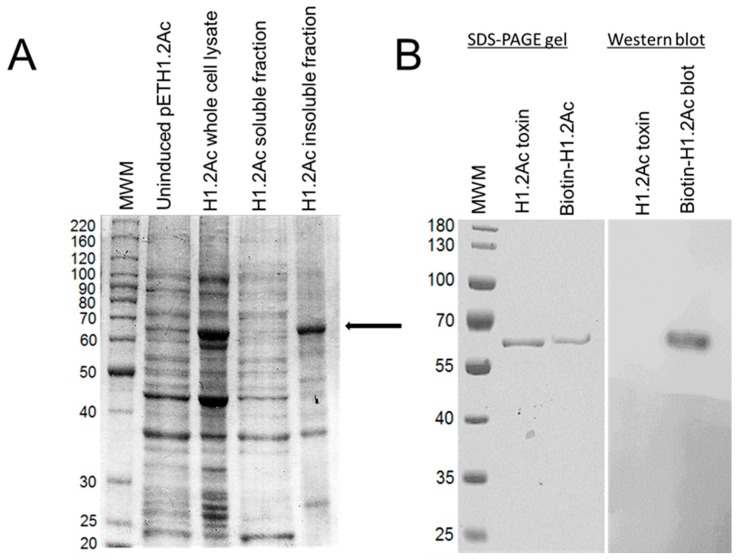
Expression, purification, and biotinylation of H1.2Ac. The arrow indicates the expected size for the H1.2Ac protoxin. (**A**) SDS-PAGE gel showing proteins expressed by transformed *E. coli* cultures with or without IPTG induction, as stated in the figure. The H1.2Ac protein was only detected in the insoluble fraction of the total cell lysate. (**B**) Electrophoretic detection of purified trypsin-activated H1.2Ac and Western blot detecting biotinylated H1.2Ac using enhanced chemiluminescence. MWM, molecular weight markers in kDa.

The H1.2Ac hybrid was over-expressed as a 68 kDa protein that included a 6x-His tag (Figure 2A). Processing by trypsin yielded an activated 60 kDa H1.2Ac protein, which was resistant to further proteolysis, as found for Cry1Ac (Figure 2B).

### 2.2. Insecticidal Activity

The toxicity of H1.2Ac against the neonates of *A. gemmatalis* and *C. includens* was tested in 7-day bioassays, only scoring for dead larvae. The H1.2Ac hybrid-activated toxin was found to be highly active against *A. gemmatalis* when compared to activity by the parental Cry1Ac and Cry2Ac7 toxins (Table 1). While the LC50 obtained for H1.2Ac was lower than for Cry1Ac or Cry2Ac7, this difference was not considered significant based on the overlapping 95% confidence intervals of the three toxins. 

Both Cry1Ac and Cry2Ac7 were active against *C. includens* neonates, although this species was about 5-fold less susceptible to the toxins than *A. gemmatalis* larvae (Table 1). In contrast to H1.2Ac activity against *A. gemmatalis*, we did not detect toxicity of H1.2Ac against the larvae of *C. includens*. Moreover, we did not detect growth inhibition of *C. includens* larvae, even at the highest H1.2Ac concentration tested (1 µg/cm^2^).

### 2.3. Toxin Binding Assays

**Figure 3:**
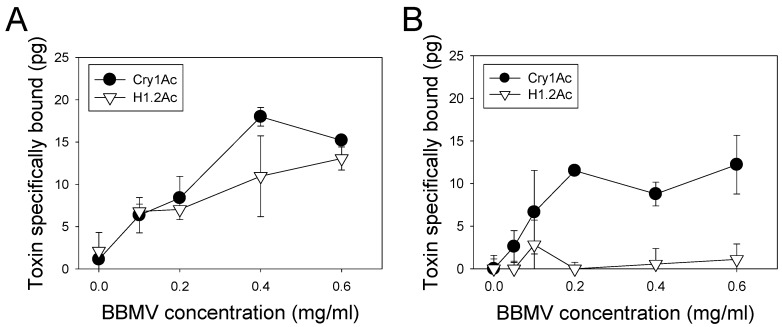
Specific binding of ^125^I-Cry1Ac (black circles) and ^125^I-H1.2Ac (inverted open triangles) to midgut brush border membrane vesicles (BBMV) of *A. gemmatalis* (**A**) and *C. includens* (**B**). Values of specific binding shown were obtained by subtracting non-specific from total binding for each BBMV concentration. Each data point and error bar represent the mean and corresponding standard error from two independent experiments performed in duplicate.

Domain III of Cry1Ac has been involved in the specificity of binding to midgut receptors [14]. Consequently, we hypothesized that the lack of H1.2Ac toxicity in *C. includens* was related to alterations in binding when compared to the parental toxins. In saturation binding assays, we monitored the binding of increasing ligand (radiolabeled Cry1Ac or H1.2Ac) concentrations, to midgut brush border membrane vesicle (BBMV) proteins from *C. includens* and *A. gemmatalis*. In the case of *A. gemmatalis* BBMV, we detected the specific binding of both Cry1Ac and H1.2Ac (Figure 3A). In contrast, while we detected binding of radiolabeled Cry1Ac to BBMV from *C. includens* (Figure 3B), we did not detect specific binding of H1.2Ac to BBMV proteins from that insect.

### 2.4. Competition Binding Assays

**Figure 4:**
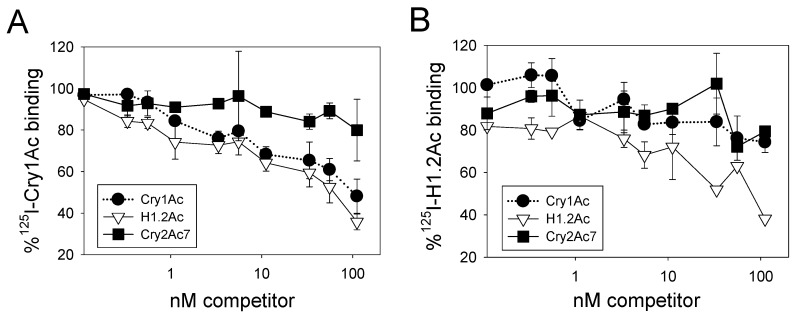
Competition of ^125^I-Cry1Ac (**A**) or ^125^I-H1.2Ac (**B**) to BBMV of *A. gemmatalis* by unlabeled Cry1Ac (black circles), H1.2Ac (inverted open triangles), and Cry2Ac7 (black squares). Each data point represents the mean and corresponding standard error from two independent experiments performed in duplicate.

Competition assays were performed to test the sharing of binding sites between radiolabeled Cry1Ac or H1.2Ac and unlabeled competitors in BBMV from *A. gemmatalis*. Binding of ^125^I-Cry1Ac was displaced by unlabeled Cry1Ac (Figure 4A), allowing the estimation of an apparent dissociation constant (*Kd*) of 0.33 ± 0.09 nM and a concentration of binding sites (*Bmax*) of 108 ± 30 nmol/mg of BBMV protein. In comparison, H1.2Ac displaced all ^125^I-Cry1Ac binding with similar affinity (*Kd* = 0.20 ± 0.04 nM) but with lower concentration of binding sites (*Bmax* = 64 ± 11 nmol/mg BBMV protein). No displacement of ^125^I-Cry1Ac binding was observed when using Cry2Ac7 as a competitor (Figure 4A).

In competition assays with ^125^I-H1.2Ac, we observed displacement of binding by unlabeled H1.2Ac, with a *Kd* of 0.20 ± 0.04 nM and a *Bmax* of 82 ± 16 nmol/mg of BBMV protein. When using Cry1Ac or Cry2Ac7 as unlabeled competitors, we detected partial (20%) displacement of ^125^I-H1.2Ac binding at the highest competitor concentration tested (Figure 4B).

### 2.5. Ligand Blots of Biotinylated Toxins

**Figure 5:**
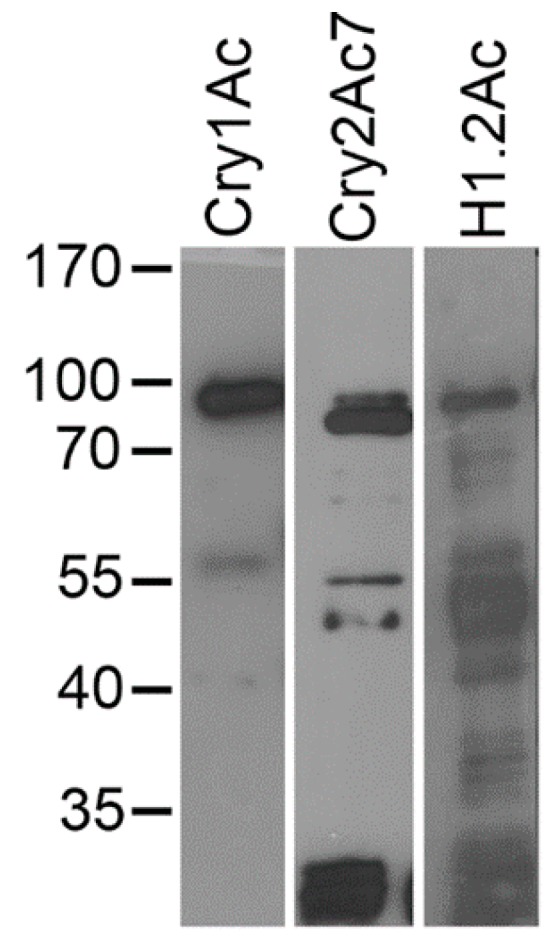
Ligand blot of midgut BBMV proteins from fifth instar *A. gemmatalis* larvae detected with biotinylated Cry1Ac, H1.2Ac, and Cry2Ac7 toxins, as indicated. The BBMV proteins were resolved by SDS-PAGE and transferred to PVDF filters, which, after blocking, were probed with the biotinylated Cry toxins. Bound toxins were detected using enhanced chemiluminescence. Molecular mass markers (in kDa) are shown at the left of the gel for reference.

Biotin-labeled Cry1Ac bound mostly to a ~90-kDa protein band in *A. gemmatalis* BBMV (Figure 5, Cry1Ac lane), but binding was also observed to a protein band of approximately 57 kDa. In the case of Cry2Ac7, the biotinylated toxin also recognized the 90-kDa protein band recognized by Cry1Ac, but it also bound to protein bands of 75-, 55-, 50-, and <30-kDa protein bands in *A. gemmatalis* BBMV (Figure 5, Cry2Ac7 lane). In contrast, the H1.2Ac toxin recognized all protein bands recognized by Cry1Ac and Cry2Ac7, in addition to interactions with protein bands between 35 and 50 kDa (Figure 5, H1.2Ac lane).

## 3. Discussion

Soybeans engineered to produce the Cry1Ac toxin (Intacta^TM^) efficiently control the larvae of *A. gemmatalis* and *C. includens* [15], yet production of a single toxin greatly increases the probability of resistance evolution in target insects. Novel insecticidal proteins could reduce the risk of resistance evolution and expand the range of activity if pyramided with Cry1Ac in transgenic soybeans. Domain III of Cry1Ac contains a lectin fold involved in binding to *N*-acetylgalactosamine (GalNAc) on putative receptors [4,14,16]. This affinity for glycosylated proteins was proposed in a sequential binding model to promote interactions of toxin oligomers with receptors, leading to toxin insertion in the cell membrane [17]. Analysis of Cry2Ac7 domain III determined that out of the N506, Q509, and Y513 amino acid residues in Cry1Ac involved in binding to GalNAc [14], only Q509 is conserved. Consequently, we designed a toxin hybrid (H1.2Ac) combining domains I and II from Cry1Ac as the most active Bt toxin against *A. gemmatalis* and *C. includens*, and domain III from Cry2Ac7 as a novel toxin with toxicity against both species but predicted not to share binding sites with Cry1Ac [18].

Previous reports of toxicity enhancement [9,19] and reduction [20,21] after alteration of domain III in Cry1Ac predicted potential effects on H1.2Ac activity. However, we unexpectedly found that while H1.2Ac displayed high activity against *A. gemmatalis*, it did not kill or delay development in the larvae of *C. includens*. Results from binding assays confirmed that this lack of H1.2Ac toxicity against *C. includens* was due to the absence of specific toxin binding to midgut proteins. Since the only region in the H1.2Ac hybrid diverging from Cry1Ac is domain III, these observations identify this domain as the main Cry1Ac binding specificity (and toxicity) determinant in *C. includens*. In contrast, insecticidal activity and displacement of Cry1Ac binding by H1.2Ac in BBMV from *A. gemmatalis* support that in this insect Cry1Ac binding specificity mostly resides in domain II, which is shared between both toxins. However, testing of alternative hybrid toxins containing domains I and II of Cry1Ac and domain III from a toxin inactive against *A. gemmatalis* would be necessary to conclude the importance of domain II for specificity in that insect. Remarkably, the concentration of binding sites in *A. gemmatalis* BBMV was higher for Cry1Ac than for H1.2Ac, which may suggest the involvement of Cry1Ac domain III in recognizing a population of binding sites. However, the fact that H1.2Ac and Cry1Ac are equally active against *A. gemmatalis* larvae supports the finding that interactions with the binding sites recognized by domain III of Cry1Ac may not be relevant to toxicity against *A. gemmatalis*. Dependence on domain III for recognition of this second population of Cry1Ac binding sites in *A. gemmatalis* BBMV would also help explain why GalNAc significantly inhibited Cry1Ac binding to BBMV from *C. includens,* while the effect was more modest on Cry1Ac binding to *A. gemmatalis* BBMV [18]. Interestingly, previous reports suggest the existence of two populations of Cry1Ac binding sites in *C. includens*, but not in *A. gemmatalis* [18]. One possibility to explain this discrepancy is that while there may be multiple populations of Cry1Ac binding sites in *A. gemmatalis* BBMV, they are recognized with similar affinity by the toxin, preventing their discrimination as distinct sites in competition-binding assays. 

As expected from only sharing one of the two putative binding specificity domains with H1.2Ac, neither Cry1Ac nor Cry2Ac7 displaced all H1.2Ac binding to *A. gemmatalis* BBMV. These results suggest that sites recognized by domain II from Cry1Ac differ from sites recognized by domain III from Cry2Ac7, as predicted from the lack of shared binding sites between Cry1A and Cry2A toxins in *A. gemmatalis* [18]. While one needs to keep in mind the limitations associated with ligand blotting [22], comparison of the pattern of *A. gemmatalis* BBMV proteins recognized by Cry1Ac, H1.2Ac and Cry2Ac7 toxins suggested sharing of some protein bands between H1.2Ac and Cry1Ac or Cry2Ac7. Further research is needed to identify the proteins present in these electrophoretic regions recognized by these toxins, and to elucidate whether H1.2Ac recognizes BBMV proteins not bound by Cry1Ac or Cry2Ac7.

Differential toxicity of H1.2Ac to *A. gemmatalis* and *C. includens* clearly supports the existence of differences in the way Cry1Ac interacts with the membrane binding sites in these insects. This observation may suggest that integrative models developed using a particular Cry toxin and insect may not be completely applicable to other species. Importantly, *A. gemmatalis* and *C. includens* differ in their susceptibility to Cry1Ac, with *A. gemmatalis* being most susceptible [13]. While speculative, recognition of binding sites that are not conducive to toxicity by domain III of Cry1Ac may help explain lower susceptibility in *C. includens* compared to *A. gemmatalis*. Thus, while in *A. gemmatalis* both domain II and III would be involved in binding to receptors, only domain III would be relevant in *C. includens*, potentially limiting the number of interactions with lethal receptors. While the present study presents evidence for the importance of Cry1Ac domain III for toxicity against *C. includens*, further research would be needed to identify lethal Cry1Ac receptors and determine their interactions with domains II and/or III of Cry1Ac. This information contributes to the design of more active insecticidal proteins against this pest and our general understanding of the Cry mode of action in Lepidoptera.

## 4. Materials and Methods

### 4.1. Cloning of H1.2Ac Hybrid

The *cry1Ac* (GenBank accession no. EU250285) and *cry2Ac7* (accession no. CAL18690.1) genes were cloned from Bt strains DNB-BtV and SBSBT-1 from the School of Biological Sciences, University of the Punjab (Lahore, Pakistan). Amplified products were inserted into the pET28a(+) expression vector (Novagen, Madison, WI, USA) using engineered restriction sites. The deduced amino acid sequences were scanned using an EMBL-EBI InterProScan tool [23] to locate the toxin domains through alignment to other structurally defined Cry toxins. The DNA sequences encoding the selected domains in the activated Cry1Ac and Cry2Ac7 toxins were amplified using the primers shown in Table 2.

The strategy to fuse the selected gene segments is shown in Figure 1. Cloning PCR reaction mixtures (50 µL) contained 1 ng of template plasmid DNA, 1X Taq buffer with (NH4)2SO4, 2 mM MgCl_2_, 0.25 mM dNTP mix, 100 pmol of each primer, and 1 U of Taq DNA polymerase (Fermentas, ThermoFisher, Waltham, MA, USA). Reaction conditions included initial denaturation at 95 °C for 5 min followed by 35 cycles of denaturation at 94 °C for 90 s, annealing at 67 °C (for *cry1Ac* template DNA) and 68 °C (for *cry2Ac7* template DNA) for 45 s, extension at 72 °C for 60 s, and then a 10 min final extension at 72 °C. The amplified DNA segments DIDIIAc and DIII2Ac were TA cloned in the pTZ57R/T vector (Fermentas) to get the DIDIIAcpTZ and DIII2AcpTZ constructs, respectively. The DIDIIAcpTZ construct and pET28a(+) plasmid were restriction digested with NdeI and SalI and ligated to generate construct 1AcDIDIIp28. After digestion with SalI and XhoI, the 1AcDIDIIp28 and DIII2AcpTZ plasmids were ligated together to generate the H1.2Ac recombinant DNA plasmid containing a terminal 6X-His tag for affinity purification of the recombinant protein. The presence and orientation of inserts was confirmed by DNA sequencing at the School of Biological Sciences, University of the Punjab (Pakistan). The predicted hybrid protein sequence was also searched as done for the parent sequences for the presence of specific domains through the InterProScan tool.

### 4.2. Over-Expression and Purification of Recombinant Protoxins

The *E. coli* BL21 CodonPlus (DE3)-RIPL competent cells were transformed with recombinant plasmids containing *cry1Ac*, *cry2Ac7* or *H1.2Ac* genes. Kanamycin-selected transformants were used to inoculate 500 mL of LB media supplemented with kanamycin (50 µg/mL final concentration). Protein over-expression was induced with 1.0 mM (Cry1Ac), 0.1 mM (Cry2Ac7) or 0.25 mM (H1.2Ac) IPTG when OD600 became ~0.8, and cultures were further incubated at 37 °C in a shaking incubator for 6–8 h. The cells were harvested by centrifugation and resuspended in 50 mM Tris-HCl, 20 mM NaCl (pH 8.8) buffer, and then lysed ultrasonically for 20 cycles of 5 s on and 55 s off at 70% amplitude in a Model 505 Sonic Dismembrator (Fisher Scientific, Hampton, NH, USA). Inclusion bodies were washed twice with 50 mM Tris-HCl, 0.2% Triton-X-100, and 0.5 M NaCl (pH 8.8). Proteins in the form of inclusion bodies were washed with 20 mM Tris-HCl (pH 9.0) and solubilized by incubation at 37 °C for 30–45 min in denaturing buffer (20 mM Tris-HCl, pH 9.0, plus 0.5 mMNaCl, 10 mM imidazole, 8 M urea, and 0.1–0.2% β-mercaptoethanol), followed by centrifugation at 15,000× *g* at 4 °C. The solubilized proteins were purified and on-column refolded using Ni-NTA Agarose affinity resin (Thermo Scientific, Hampton, NH, USA) according to the manufacturer’s instructions. Briefly, 1.5 mL resin mixture was washed with 6 mL distilled water and then equilibrated with 3 mL of denaturing binding buffer (20 mM Tris-HCl, pH 9.0, plus 0.5 mM NaCl, and 8 M urea) for 2 min. Binding of denatured proteins with agarose resin was achieved by incubation for 1 h at room temperature on a rotatory shaker. The protein-bound resin was packed in a 10 mL purification column under gravity and washed with 5 mL denaturing binding buffer to remove unbound proteins. The bound proteins were refolded on-column by washing with 5 mL of denaturing wash buffer with a decreasing step gradient of urea (6 M, 4 M, and 2 M urea). The column was then washed twice with 8–10 mL of native buffer (20 mM Tris-HCl, 0.5 mM NaCl, pH 9.0) followed by 15–20 mL of elution buffer (250 mM imidazole in native buffer). Elution of Cry1Ac was detected at 60% (150 mM imidazole) elution buffer, while 100% (250 mM imidazole) was needed to elute the H1.2Ac protein. The fractions were analyzed on SDS-10% agarose gel and dialyzed against 20 mM Tris-HCl, (pH 8.8), 0.1 M NaCl.

### 4.3. Activation and Purification of the Recombinant Toxins

Protoxins purified with Ni-NTA chromatography were quantified using the Qubit Protein Assay kit in a Qubitfluorometer (Life Technologies, Carlsbad, CA, USA), and then activated by incubation with a 5:1 *w*/*w* ratio of toxin to TPCK-treated trypsin (Sigma-Aldrich, St. Louis, MO, USA) at 37 °C for 75 min (Cry1Ac and H1.2Ac) or 90 min (Cry2Ac7). Activation was confirmed by SDS-10% PAGE and activated toxins were dialyzed against 20 mM Na_2_CO_3_ (pH 9.8) buffer. The activated protein was further purified by anion exchange chromatography on a Hi Trap Q HP column (GE Healthcare Life Sciences, Pittsburgh, PA, USA) connected to an ÄKTA pure FPLC (GE Healthcare Life Sciences, Pittsburgh, PA, USA), as described elsewhere [24]. 

### 4.4. Insect Bioassays

Eggs of *A. gemmatalis* and *C. includens* were purchased from Benzon Research (Carlisle, PA, USA), and neonate larvae were used for bioassays against active toxins. Freshly prepared general purpose Lepidoptera diet (BioServ) was solidified in 128-well trays and surface contaminated with 75 µL of test solution per well. Toxin buffer (50 mM Na_2_CO_3_, pH 9.8) was used as a control and to dilute the test proteins. After the adsorption of the solution and drying, one neonate was placed per well and the wells covered with adhesive porous plastic sheets. Bioassay trays were incubated at 70–80% humidity and 14:10 L:D photoperiod at 27(±1)°C. Sixteen larvae were assayed per concentration per replicate, and bioassays were performed in duplicate and replicated thrice. Dead larvae were scored after 7 days and Abbott’s formula [25] was applied to correct for natural mortality. POLO-PLUS software [26] was used for calculation of LC50s (50% lethal concentrations) using probit analysis.

### 4.5. Preparation of Brush Border Membrane Vesicles

Untreated larvae of *A. gemmatalis* and *C. includens* were reared under the same conditions described for bioassays until fifth instar, and then their midgut dissected and instantly frozen on dry ice and stored at −80 °C for less than a week. Brush border membrane vesicles (BBMVs) were prepared as described elsewhere [27], with minor modifications [28]. Enrichment of aminopeptidase-N activity was assayed to determine the purity of BBMV preparations using leucine ρ-nitroanilide as substrate, as described elsewhere [16]. The aminopeptidase activities were 4–7 times higher in the final BBMV preparation compared to initial crude midgut homogenates. BBMV proteins were quantified with the Qubit fluorometer as above and stored at −80 °C until used.

### 4.6. Radiolabeling and Biotinylation of Cry Proteins

Purified activated H1.2Ac and Cry1Ac toxins were radiolabeled using the chloramine T method as described elsewhere [24]. Radioiodination of proteins was confirmed by counting radioactivity in a Wizard^2^ gamma counter (Perkin Elmer, Waltham, MA, USA) and autoradiography. Specific activities of the radiolabeled toxins were 2.23 mCi/pmol for Cry1Ac and 1.71 mCi/pmol for H1.2Ac. 

Purified H1.2Ac, Cry1Ac, and Cry2Ac7 toxins were biotinylated using 30 nM of EZ-Link NHS-LC-Biotin (Thermo Scientific, Hampton, NH, USA) in phosphate buffered saline (PBS) buffer (137 mM NaCl, 2.7 mM KCl, 1.8 mM KH_2_PO_4_, 10 mM Na_2_HPO_4_, pH 7.4) as described elsewhere [29]. Labelled proteins were quantified and stored at −80 °C until used.

### 4.7. Binding and Competition Assays 

The optimal concentration of *A. gemmatalis* and *C. includens* BBMV for use in competition experiments was determined from BBMV binding assays, as described elsewhere [30]. Briefly, 1.0 nM ^125^I-H1.2Ac or ^125^I-Cry1Ac was incubated for 1 h at room temperature with BBMV proteins (from 0 to 0.6 mg/mL) in binding buffer (PBS pH 7.5 plus 0.1% BSA) in a final reaction volume of 0.1 mL. Reactions were stopped by centrifugation and pellets washed once with 0.5 mL of ice-cold binding buffer before measuring radioactivity in a Wizard^2^ gamma counter (Perkin Elmer, Waltham, MA, USA). Non-specific binding was determined in reactions containing a 500-fold excess of unlabeled homologus toxin. Specific binding was determined by subtracting non-specific from total binding. Each experiment was performed in duplicate and replicated twice.

In competition binding assays, BBMV proteins (20 µg) of *A. gemmatalis* were incubated with 1 nM ^125^I-H1.2Ac or ^125^I-Cry1Ac in the presence of increasing concentrations of an unlabeled competitor at room temperature in a final reaction volume of 0.1 mL. Reactions were stopped after 1 h by centrifugation and washing as described above, and then radioactivity measured in the final pellets. The amount of radiolabeled toxin bound in the absence of the competitor was considered as 100% binding in determining the percentage of radiolabeled toxin remaining bound in the presence of the competitor. Competition data from two replicated experiments performed in duplicate for each toxin were pooled and analyzed using SigmaPlot v.11.0 software to obtain the apparent dissociation constant (*Kd*) and concentration of binding sites (*Bmax*).

### 4.8. Ligand and Western Blotting

Ligand blots were performed as described elsewhere [29]. Briefly, BBMV proteins (30 µg) from *A. gemmatalis* or *C. includens* were resolved by SDS-10%PAGE and transferred to PVDF filters. After blocking in PBS pH 7.5, 0.1% Tween-20, and 3% BSA for 1 h., filters were probed with biotinylated H1.2Ac (0.16 nM), Cry1Ac (0.5 nM), or Cry2Ac7 (0.50 nM) for 1 h. The membranes were then washed with PBS pH 7.5, 0.1% Tween-20, and 0.1% BSA and then probed with a 1:15,000 dilution of streptavidin-HRP (Thermo Scientific, Hampton, NH, USA) for an hour. After washing as above, the bound toxins were visualized using enhanced chemiluminescence (SuperSignal West Pico substrate; Thermo Scientific, Hampton, NH, USA). Materials and Methods should be described with sufficient detail to allow others to replicate and build on published results.

## Figures and Tables

**Table 1:** Toxicity of hybrid activated proteins against the neonates of *A. gemmatalis* and *C. includens*. LC50 ^a^ (95% fiducial limits). Bioassays with both parental toxins and the H1.2Ac hybrid were performed simultaneously.

Toxin	*A. gemmatalis*	*C. includens*	Source ^b^
H1.2Ac	10.20 (1.57–20.80)	NA ^c^	this study
Cry1Ac	20.31 (11.09–27.90)	109.48 (52.85–179.50)	[13]
Cry2Ac7	46.53 (5.70–102.60)	214.18(109.71–315.91)	[13]

^a^ Protein concentration in ng/cm^2^. ^b^ LC50 values of Cry1Ac and Cry2Ac7 are referenced here for comparison. Bioassays with all three toxins were performed simultaneously. ^c^ Not available, as no toxicity was detected.

**Table 2:** Primers used for PCR amplification of selected domains of Cry toxins with restriction sites underlined and melting temperatures (T_m_) in °C.

Primer	Sequence (5′-3′)	Restriction Enzyme	T_m_
1AcFD1	TGGAGGTCATATGGATAACAATCCGAACATC	NdeI	69.7
1AcRD2	GTAGTCGACTTCAGCACTACGATGTATCCA	SalI	70.8
2AcFD3	TCTGTCGACTTCACCGTATCTCCAATACATGCC	SalI	73.0
2AcRD3	TGTCTCGAGTTAATACAGTGGTGGAAGGTTAG	XhoI	71.3
